# Detecting malingering mental illness in forensics: Known-Group Comparison and Simulation Design with MMPI-2, SIMS and NIM

**DOI:** 10.7717/peerj.5259

**Published:** 2018-07-25

**Authors:** Barbara De Marchi, Giulia Balboni

**Affiliations:** 1 Centro Ferrarese di Neuropsichiatria, Neuropsicologia e Riabilitazione, Ferrara, Italy; 2 Department of Philosophy, Social and Human Sciences and Education, University of Perugia, Perugia, Italy

**Keywords:** Malingering, Forensics, Psychometric tests, Psychopathology

## Abstract

**Background:**

Criminal defendants may often exaggerate psychiatric symptoms either to appear non-accountable for their actions or to mitigate their imprisonment. Several psychometric tests have been proposed to detect malingering. These instruments are often validated by Simulation Design (SD) protocols, where normal participants are explicitly requested to either simulate a mental disorder or respond honestly. However, the real scenarios (clinical or forensic) are often very challenging because of the presence of genuine patients, so that tests accuracy frequently differs from that one obtained in well-controlled experimental settings. Here we assessed the effectiveness in criminal defendants of three well-known malingering-detecting tests (MMPI-2, SIMS and NIM) by using both Known-Group Comparison (KGC) and Simulation Design (SD) protocols.

**Methods:**

The study involved 151 male inmates. Participants to the KGC protocol were all characterized by a positive psychiatric history. They were considered as genuine patients (KGC_Controls) if they had some psychiatric disorders already before imprisonment and scored above the cutoff of SCL-90-R, a commonly used test for mental illness, and as suspected malingerers (KGC_SM) if they were diagnosed as psychiatric patients only after imprisonment and scored below the SCL-90-R cutoff. Participants to SD protocol had no history of psychiatric disease and scored below the SCL-90-R cutoff. They were randomly assigned to either group: Controls (requested to answer honestly, SD_Controls) and simulated malingerers (requested to feign a psychiatric disease, SD_SM). All participants were then submitted to MMPI-2, NIM and SIMS.

**Results:**

Results showed that while MMPI-2, SIMS and NIM were all effective in discriminating malingerers in the SD, SIMS only significantly discriminated between KGC_Controls and KGC_SM in the Known-Group Comparison. Receiver Operating Characteristic (ROC) curves analysis confirmed the better sensitivity of SIMS with respect to the other tests but raised some issues on SIMS specificity.

**Discussion:**

Results support the sensitivity of SIMS for the detection of malingering in forensic populations. However, some specificity issues emerged suggesting that further research and a good forensic practice should keep into account multiple measures of malingering, including psychometric data, clinical and social history and current clinical situation. These methodological constraints must be kept in mind during detection of malingering in criminal defendants reporting psychiatric symptoms.

## Introduction

The detection of malingering is an important topic in psychology (for a comprehensive review, see Seron, 2014) raising practical and ethical issues (Bass & Halligan, 2014). A significant percentage of individuals undergoing psychological evaluation may feign psychopathological symptoms especially when the context is perceived as challenging, such as in the forensic (Rogers, Sewell & Goldstein, 1994) and clinical (Noeker & Petermann, 2011) settings. The forensic framework and in particular criminal defendants represent a challenging situation characterized by a high frequency of malingering behaviors (Merten & Rogers, 2017) although with different base rate (Mittenberg et al., 2002; Young, 2014). Criminal defendants simulate psychopathology to avoid or to delay punishment or to obtain more favorable conditions (Rogers, 2008). Because of these reasons, over the past two decades, symptom validity research in this field has significantly intensified (Otto & Heilbrun, 2002; Douglas, Otto & Borum, 2003; McLaughlin & Kan, 2014).

Various studies have demonstrated that several psychopathologies can be simulated by malingering individuals (Lees-Haley & Dunn, 1994; Bowen & Bryant, 2006; Baity et al., 2007). They include major depression, generalized anxiety disorder and post-traumatic stress disorder (PTSD). Several psychometric instruments have been proposed to detect malingering, from multidimensional personality inventories (Berry et al., 2001; Edens, Cruise & Buffington-Vollum, 2001) to specific tests for malingering (for a review, see Sellbom & Bagby, 2008).

In the present study we selected three tests used for malingering detection: The Minnesota Multiphasic Personality Inventory (MMPI)-2 (Butcher et al., 1989), the Structured Inventory of Malingered Symptomatology (SIMS) (Smith & Burger, 1997; Widows & Smith, 2005) and the Negative Impression Management (NIM) scale of the Personality Assessment Inventory (PAI) (Morey, 1991) based on the existence of Italian versions for MMPI-2 (Pancheri & Sirigatti, 1995) and SIMS (La Marca et al., 2012) and of an Italian translation of the NIM specifically done for the present study (see ‘Methods’ Section).

These three instruments have been selected because of two main reasons. The first one was their diffusion. Indeed, according to an analysis of the literature on malingering detection in forensics, MMPI-2, SIMS and NIM were among the most used instruments at the moment of data collection. The second reason was the existence of an Italian version of the instrument.

MMPI-2 is a personality questionnaire that enables the detection of several psychopathological dimensions. Additionally, by means of specific validity scales, MMPI-2 can be used to detect inconsistent responding, over-reporting and under-reporting psychological symptoms, even in healthy participants. These reasons have expanded the use of the MMPI-2 outside the clinical environment, from the selection of workers and employees to the detection of fraudulent behaviors in people asking financial compensations and to prisoners and criminal defendants (Pope, Butcher & Seelen, 2006). In particular, as far as criminal defendants are concerned, the family of F (Infrequency) scales provides most information about simulation behaviors (Meehl & Hathway, 1946) being made up of items very rarely endorsed by the MMPI normative group (for a discussion, see Rogers, 1997a; Rogers, 1997b). This strategy has been extended to the MMPI-2 with the creation of the Fb (Infrequency Back), the Fp (Infrequency Psychopathology; Arbisi & Ben-Porath, 1995), and the Fc (Criminal Offender Infrequency; Mergargee, 2004; Mergargee, 2006) scales. More recently, other scales have been developed to detect malingering behaviors in non-criminal settings (Fake Bad Scale, FBS; Response Bias Scale, RBS; for a recent review, see Merten & Rogers, 2017).

MMPI-2 is, however, very long to administer. For this reason, clinicians frequently prefer faster and easier screening measures, particularly useful when the assessment has to be conducted in large participants cohorts in particular for preliminary screenings (Edens, Poytress & Watkins-Clay, 2007). The NIM scale of the PAI (Morey, 1991), has been considered to provide an adequate level of malingering detection (Morey & Lanier, 1998). Indeed, NIM scale has been included in the PAI as a measure of exaggeration or malingering and consists of items rarely endorsed in the case of significant diseases and items associated with a very negative description of participants themselves. Studies of the NIM scale have shown different effectiveness cutoff values. Some authors (Kucharski et al., 2007) have reported that NIM scale was very effective in discriminating between malingering and genuine patients inmates, previously classified by the Structured Interview of Reported Symptoms (SIRS; Rogers, Bagby & Dickens, 1992).

The Structured Inventory of Malingered Symptomatology (SIMS) (Smith & Burger, 1997) is another test which has been demonstrated to achieve an acceptable level of accuracy in discriminating between malingering and honestly responding individuals (Smith, 1993). However, the SIMS test seems less effective when applied to “sophisticated” simulators (e.g., graduate students in psychology) than in the case of malingerers lacking a high level of education (Vitacco et al., 2007). Some authors, however, support the effectiveness of the SIMS across genders, cultures and languages and its low reading level, that make it available to a wide range of individuals (Alwes et al., 2008; Smith, 2008).

The majority of the works aiming at validating malingering detecting instruments in forensics adopt the so-called Simulation Design (SD) paradigm (see Sellbom et al., 2010). In SD, participants assigned to the experimental group are explicitly requested to answer by simulating a psychiatric illness while control participants are asked to answer honestly.

A different approach to evaluate the validity of malingering detection instruments is represented by the Known-Group Comparison (KGC) paradigm, where participants are previously subdivided into cathegories (suspected simulators and supposed sincere answerers) by using some supposedly independent criterion such as the Structured Interview of Reported Symptoms (SIRS), a largely used test for detecting simulation behaviors (Rogers, 1986; Rogers, Bagby & Dickens, 1992) or indications of the psychiatric staff (Edens, Poytress & Watkins-Clay, 2007). The KGC paradigm has been used to evaluate MMPI-2 (Bocaccini, Murrie & Duncan, 2006; Barber-Rioja et al., 2009; Toomey, Kucharski & Duncan, 2009), NIM (Rogers et al., 1998; Mogge & LePage, 2004; Bocaccini, Murrie & Duncan, 2006; Gaines et al., 2007) and SIMS (Lewis, Simcox & Berry, 2002; Edens, Poytress & Watkins-Clay, 2007; Vitacco et al., 2007; Clegg, Fremouw & Mogge, 2009). However, SIRS has been more recently shown to be a classification criterion less independent than expected because its outcome significantly correlates with the above mentioned tests (Laffon, 2009; Sellbom et al., 2010).

In the present study we investigated the effectiveness of MMPI-2, NIM and SIMS in detecting malingering behaviors using both KGC and SD paradigms. Participants to KGC were all criminal defendants with a diagnosis of psychiatric disease after imprisonment. They were considered as genuine patients (KGC_Controls) if they did show psychiatric symptoms already before imprisonment and scored above the cutoff of the Symptom Check List-90-Revised (SCL-90-R, a commonly used test for mental illness; Derogatis, Lipman & Covi, 1973). As suspected malingerers (KGC_SM) we considered those inmates lacking any psychiatric history before imprisonment, diagnosed as psychiatric patients only after it and scoring below the SCL-90-R cutoff. Participants to SD protocol had no history of psychiatric disease and scored below the SCL-90-R cutoff. They were randomly assigned to either group: Controls (requested to answer honestly, SD_Controls) and simulated malingerers (requested to feign a psychiatric disease, SD_SM). All participants were then submitted to MMPI-2, NIM and SIMS.

## Methods

### Participants

Participants (*n* = 151, mean age 39.3 ± 11 (SD), range 22–72) were male inmates of Penitentiary Institutes of the North of Italy, voluntarily participating to the study after providing their written informed consent. The experimental procedure was approved by the Institutional Review Board of the Department of Penitentiary Administration of the Piedmont and Valle D’Aosta Region (approval n. 49720/09). All the procedures complied with the Helsinki Declaration of Ethical Principles for Medical Research. Participants, anonymity was fully preserved.

All participants were submitted to SCL-90-R, a self-report questionnaire designed to assess the presence and the severity of psychological symptoms during the last week before test administration (Derogatis, Lipman & Covi, 1973; Italian adaptation by Sarno et al., 2011).

There were two different experimental designs: KGC and SD (see Table 1). The KGC design involved defendants with a diagnosis of psychiatric illness after imprisonment (DSM IV-TR Axis I disorders, see Table 1 and Table 2). They were assigned to either two categories on the basis of the following criteria: inmates diagnosed only after imprisonment and scoring below the SCL-90-R cutoff were classified as Suspected Malingerers (KGC_SM). Inmates having a positive psychiatric history already before imprisonment and scoring above the SCL-90-R cutoff (*T* value ≥ 55 for at least two primary symptom dimensions or for at least one global distress index) were considered as honest responders (KGC_Controls).

**Table 1:** Criteria used to assign participants to the four groups of the present study.

	Known-Group Comparison (KGC)	Simulation Design (SD)
Controls	*n* = 35 –Mental disorders (Axis I) diagnosed *before and after* imprisonment –*Above* SCL-90-R cutoff –Requested to respond honestly	*n* = 36 –No history of mental disorders, free from psychoactive drugs –Below SCL-90-R cutoff –Requested to respond honestly
Malingerers (SM)	*n* = 22 –Mental disorders (Axis I) diagnosed *only after* imprisonment –Below SCL-90-R cutoff –Requested to respond honestly	*n* = 35 –No history of mental disorders, free from psychoactive drugs –Below SCL-90-R cutoff –Requested to simulate mental illness

**Notes.**

Axis I refers to DSM IV-TR classification (American Psychiatric Association, 2000). The names of the groups of participants adopted in the text arise from the combination of those used in this Table: KGC_SM, suspected malingerers; SD_SM, simulated malingerers; KGC_Controls, genuine patients; SD_Controls, controls of the SD requested to respond honestly. For clarifications, see text.

**Table 2:** Socio-demographic data of the participants.

	Known-Group Comparison		Simulation Design	
	KGC_SM (*n* = 22)	KGC_Controls (*n* = 35)	SD_SM (*n* = 35)	SD_Controls (*n* = 36)
**Age: mean±SD**	36 ± 10	38 ± 8	44 ± 10	41 ± 11
**Education level: *n* (%)**				
> = High school	2 (9)	1 (3)	3 (9)	5 (14)
Middle school	14 (64)	30 (86)	20 (57)	23 (64)
Elementary school	6 (27)	4 (11)	12 (34)	8 (22)
**Nationality: *n* (%)**				
Italy	10 (43)	20 (57)	*29 (80)*	*15 (42)*
Europe	4 (17)	1 (3)	*3 (11)*	*7 (19)*
Extra-Europe	8 (39)	14 (40)	*3 (9)*	*14 (39)*
**Type of Crime: *n* (%)**				
Personal injuries	5 (23)	5 (14)	*7 (20)*	*6 (17)*
Drugs	10 (45)	18 (52)	*22 (63)*	*13 (36)*
Property Crimes^*^	7 (32)	12 (34)	*6 (17)*	*17 (47)*
**Judicial status: *n* (%)**				
Condemned	2 (9)	1 (3)	3 (9)	5 (14)
Waiting for trial	20 (91)	34 (97)	32 (91)	31 (86)
**Psychiatric disease: *n* (%)**				
OCD	3 (14)	1 (3)	–	–
Anxiety Disorders	12 (55)	27 (77)	–	–
Sleep Disorders	3 (14)	2 (6)	–	–
Adaptation Disorders^**^	3 (14)	3 (9)	–	–
Psychotic Disorder	1 (5)	2 (6)	–	–

**Notes.**

Differences between simulators and controls for both KGC and SD paradigms were tested by One-way ANOVA (Age) and Fisher’s Exact Test (the remaining data). Significance threshold was set at *p* < 0.05. Numbers in italics identify significant differences between the two groups within the same experimental design. Significant differences for Nationality and Type of Crime are present in the SD group only (see text for a discussion). All participants were fluent in Italian and fully understood the questions of the questionnaires. This ruled out the possibility of a significant bias due to cultural aspects (Correa, 2010).

OCD Obsessive Compulsive Disorder * fraud, theft, robbery ** Adaptation Disorders include clinically significant emotional or behavioral symptoms that develop in response to one or more identifiable psychosocial stressors KGC_SM suspected malingerers SD_SM simulated malingerers

It should be clarified that being diagnosed after imprisonment and not scoring high on a self-measure of psychiatric symptomatology does not indicate *per se* a situation of malingering. However, psychiatric diagnoses in correctional institutes are often obtained in a quite easy way, particularly when the inmates report symptoms concerning anxiety or depression which usually lead to the prescription of largely distributed drugs (i.e., antidepressants). In our work we decided to create the “suspected malingerers” group by selecting a very specific subgroup of inmates characterized by the absence of any psychiatric illness before imprisonment, negative at the SCL-90-R test and therefore considered as psychiatric patients only because of a positive diagnosis obtained while in prison. Conversely, honest responders (KGC_Controls) were participants with a personal history of psychiatric illness already assessed before imprisonment and positive at SCL-90-R test. It may be argued that suspected malingerers had no reasons to feign at the tests even if they did it after imprisonment to gain some benefit. In our view, when an individual starts a malingering behavior it becomes an adaptive model of behavior. Moreover, all participants were unaware about the purpose of the study.

Participants to the SD paradigm were inmates with no history of psychiatric symptoms, scoring below the cutoff on the SCL-90-R. They were randomly assigned to either two groups. Participants of the first group (Simulated Malingerers, SD_SM) were asked to simulate a psychiatric illness in answering the tests. Those of the second group (SD_Controls) were requested to answer honestly. For a summary of the experimental/control groups see Table 1.

### Instruments

#### SCL-90-R

The scale Symptom Check List-90 Revised (Derogatis, Lipman & Covi, 1973) is a self-report questionnaire designed to assess the presence (and the severity) of psychiatric symptoms during the last week before the test. The test consists of 90 items, five-step responses from 0 (not at all) to 4 (very much), that identify nine primary symptom dimensions (somatization, obsessive-compulsive, interpersonal sensitivity, depression, anxiety, hostility, phobic anxiety, paranoid ideation and psychoticism) and three global distress indexes (GSI, PSDI and PST). Participants were considered positive at the test if they scored ≥ 55 (cutoff *T* value) for at least two dimensions or for at least one global distress index. The coefficients of internal consistency for the nine clinical scales were satisfactory (Derogatis, Rickels & Rock, 1976; Horowitz et al., 1998) as well as the test-retest reliability (Derogatis, Rickels & Rock, 1976).

#### MMPI-2

The MMPI-2 (Butcher et al., 1989; Italian version by Pancheri & Sirigatti, 1995) is a multi-dimensional test of psychological assessment that identifies the pathological personality traits. It consists of 567 dichotomous items (true/false) divided into a series of clinical and validity scales. The Fp (Infrequency Psychopathology) scale consists of 27 items endorsed by less than 20% of both hospitalized psychiatric patients and MMPI-2 normative group. The Fc (Criminal Offender Infrequency) scale is composed of 51 items endorsed by less than 15.5% individuals in Mergargee’s (2004; 2006) correctional sample and by less than 15% of the MMPI-2 normative sample. More recently, after verifying that non-malingering inmates often elevated both the F and Fp scales likely because of their peculiar condition, Mergargee (2004; 2006) developed the Criminal Offenders Infrequency (Fc) scale which included items rarely endorsed by criminal offenders (Barber-Rioja et al., 2009). In the present work, we evaluated Fp and Fc validity scales only, being the most sensitive scales of MMPI-2 for malingering detection in criminal defendants.

#### NIM

The Negative Impression Management scale of the Personality Assessment Inventory (Morey, 1991) is composed of 9 items that are frequently endorsed by participants having a very negative image of themselves. NIM is characterized by high test-retest reliability (Morey, 1991; Boyle & Lennon, 1994; Rogers et al., 1995) and by an adequate construct validity (Morey, 1996). In this work, we administered an Italian translation of the NIM authorized by the Author.

#### SIMS

The Structured Inventory of Malingered Symptomatology (Smith & Burger, 1997, Italian version by La Marca et al., 2012) is a psychometric instrument for malingering detection in people with at least a primary school education level. Several studies have demonstrated a high-degree of construct validity (Smith & Burger, 1997; Poytress, Edens & Watkins, 2001; Merckelbach & Smith, 2003).

### Procedure

Before the evaluation, a numbered form with socio-demographic data (nationality, education level, type of crime, judicial status, psychiatric history, eventual medications, proficiency in Italian language) was filled for each participant. The participant’s name was then written on a separate sheet of paper stapled on each numbered form. They were then asked to answer to the questions of the SCL-90-R test. Few days after, on the basis of their psychiatric history and of the results of the SCL-90-R, participants were assigned to the categories of the two experimental designs: SD (participants with a negative psychiatric history, negative at the SCL-90-R) and KGC (participants with a positive psychiatric history, either positive or negative at the SCL-90-R). SD participants were randomly assigned to either the SD_SM or the SD_Control groups. KGC participants were subdivided into two subgroups on the basis of the combination between psychiatric history and SCL-90-R results (see Table 1). At this stage, however, participants were not told about their assignment to avoid any exchange of information between them. The day of tests administration, each participant was given an envelope containing a copy of MMPI-2, SIMS and NIM tests and a pencil. A piece of paper with the same identification number of the socio-demographic form was stapled to the envelope. The sequence of the three tests was counterbalanced among participants to mitigate order effects. Participants assigned to the group of SD_Simulated Malingerers were individually told (separately from the others) to fill the tests as if they were a psychiatric patient. Participants assigned to the remaining three groups (SD_Controls, KGC_Controls and KGC_Suspected Malingerers) were individually told to always answer honestly. The time to fill the questionnaires was approximately 2 h, without any time constraint imposed on participants. At the end of the procedure, the piece of papers with the name previously stapled to the socio-demographic form was destroyed and every test was associated to the specific socio-demographic form according to the numerical code only.

Twenty-three participants were excluded from the analysis because they did not fit the grouping criteria: for the KGC design, three participants getting a psychiatric diagnosis only after imprisonment were positive at SCL-90-R and 16 participants having a double psychiatric diagnosis (before and after imprisonment) were negative at SCL-90-R. For the SD, four participants were positive at SCL-90-R. The final number of participants was therefore 128, divided into the four groups according to the criteria shown in Table 1.

### Statistical analysis

Statistical analyses were performed by Statistica 8.0 (Stat Soft Inc., USA). Demographic data were submitted to both one-way ANOVA (for age) and Fischer’s exact test (remaining data). Correlations between malingering measures (SIMS, NIM, Fp and Fc scales of MMPI-2) and SCL-90-R were calculated by Pearson’s r. Group differences were assessed by ANOVA performed on tests scores. Post hoc analyses (Newman Keuls) were performed only when factors/interactions were significant at the ANOVA. Cohen’s *d* effect size (Cohen, 1988) was determined by using the tool at: https://www.uccs.edu/lbecker/ Receiver Operating Characteristics (ROC) analysis was performed with SPSS 20 (IBM Corp., USA).

## Results

Table 2 shows the main socio-demographic characteristics of the participants to the study. Note that the most represented psychiatric symptoms among KGC participants were anxiety-related disorders. Severe diseases (i.e., psychosis) are indeed pretty rare in normal correctional institutes, as severe psychiatric patients are often found non-guilty because of their insanity and are detained in special psychiatric correctional institutes. For the SD only, some significant difference emerged between SD_SM and SD_Controls for Nationality and Type of Crime.

Table 3 displays the correlations among malingering detecting measures and between these measures and the SCL-90-R. Correlations were computed on all participants in order to take into consideration the whole spectrum of possible behaviors. At this stage, in fact, a subdivision into experimental groups would have reduced the statistical power and would have been outside the aim of this analysis. From the table, it emerged a considerable variability of the correlation values among the scales (values ranging from *r* = 0.38 to *r* = 0.89). More in detail, SIMS better correlated with NIM and, among MMPI-2 indexes, the Fc scale was the one with higher correlation with both NIM and SIMS. The value of correlation between Fp and Fc was very high (*r* = 0.89) as well as the correlation between SIMS and NIM (*r* = 0.73). It is important to note the poor correlation between the instruments used for malingering detection and the SCL-90-R, which (together with the psychiatric diagnosis criterion) was used to categorize genuine patients and suspected malingerers in the KGC paradigm. Even though other authors propose the use of SCL-90-R as an indicator of malingering (Sullivan & King, 2010), the lack of correlations shown by our results supports the use of SCL-90-R as possible external, independent criterion for grouping subjects in the KGC design.

**Table 3:** Scales intercorrelation for the whole sample (*n* = 128).

	NIM	Fp	Fc	SCL_90R
SIMS	0.73^*^	0.51^*^	0.62^*^	−0.14
NIM		0.38^*^	0.51^*^	−0.12
Fp			0.89^*^	−0.13
Fc				−0.12

**Notes.**

Raw scores were used for each scale.

TITLE Fp Infrequency Psychopathology scale Fc Infrequency Criminal Offender scale SIMS Structured Inventory of Malingered Symptomatology scale NIM Negative Impression Management scale

Asterisks indicate statistically significant correlations (*p* < 0.05).

**Figure 1:**
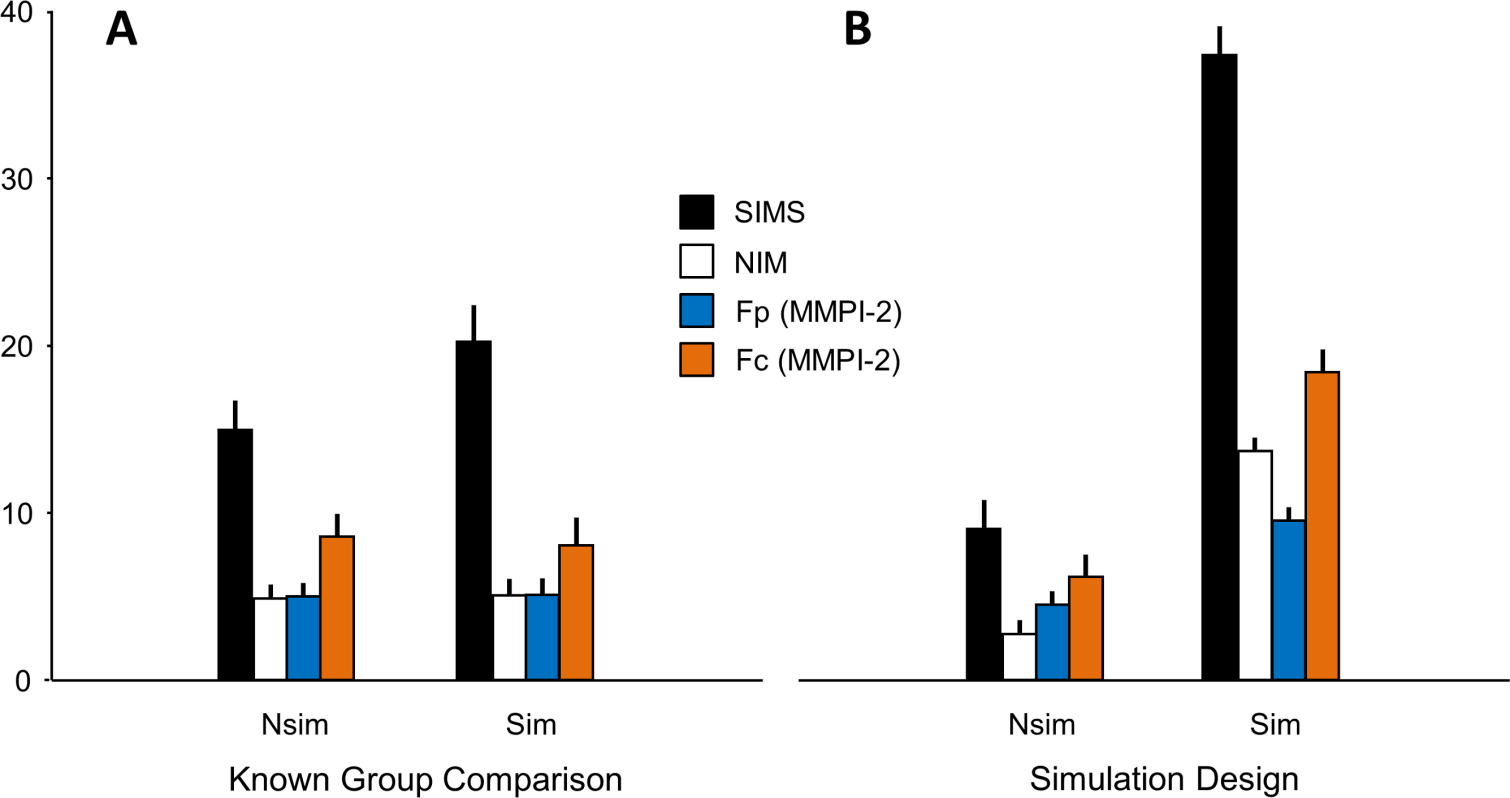
Mean scores ± SEM for SIMS, NIM, Fp and Fc psychometric tests. Nsim and Sim refer to the two groups of the Know Group Comparison (A) and Simulation Design (B) paradigms (see text). Ordinate: tests scores.

Table 4 and Fig. 1 show the main results for both KGC and SD protocols. From figure inspection it emerges the different sensitivity of the instruments in discriminating malingerers from controls in the two investigated protocols, with clear differences for all the tests in the SD but with evident differences for the SIMS only in the KGC. An ANOVA performed on individual scales (SIMS, NIM, Fp and Fc) using Design (KGC, SD) and Malingering (SM, Controls) as factors showed the significance of both of them (Design, *F*(4, 121) = 6.2, *p* = 0.0001; Malingering, *F*(4, 121) = 26.4), *p* = 0.0001) and the significance of the interaction between the two (Design × Malingering, *F*(4, 121) = 17.8, *p* = 0.0001). Post-hoc analysis (Newman Keuls, *p* < 0.05) revealed that while all the four instruments were highly sensitive in discriminating simulated malingerers (SD_SM) from controls (SD_Controls) in the SD protocol, only SIMS reached the statistical significance in discriminating between suspected malingerers (KGC_SM) from controls (KGC_Controls) (*p* = 0.01) in the KGC protocol.

**Table 4:** Mean scores ± SD of the four scales assessed in the two experimental designs.

	Known-Group Comparison			Simulation Design		
	Suspected malingerers	Controls	Cohen’s *d*	Simulated malingerers	Controls	Cohen’s *d*
SIMS	20.4 ± 11.3^*^	15.1 ± 11.9^*^	0.43	37.5 ± 8.8^*^	9.2 ± 5.8^*^	3.80
NIM	5.2 ± 4.0	5.0 ± 4.9	0.04	13.8 ± 4.6^*^	2.9 ± 2.9^*^	2.83
Fp	5.2 ± 4.5	5.1 ± 3.7	0.02	9.7 ± 5.4^*^	4.6 ± 2.1^*^	1.24
Fc	8.2 ± 8.7	8.7 ± 6.7	0.06	18.5 ± 8.9^*^	6.3 ± 4.4^*^	1.74

**Notes.**

Asterisks indicate the presence of a statistically significant difference (*p* < 0.05) at the post-hoc analysis (Newman Keuls). For other abbreviations, see text. Cohen’s *d* measures the size of the effect. Values are considered as negligible (<0.20), small (0.20–0.49), medium (0.50–0.79), or large (≥0.80).

**Figure 2:**
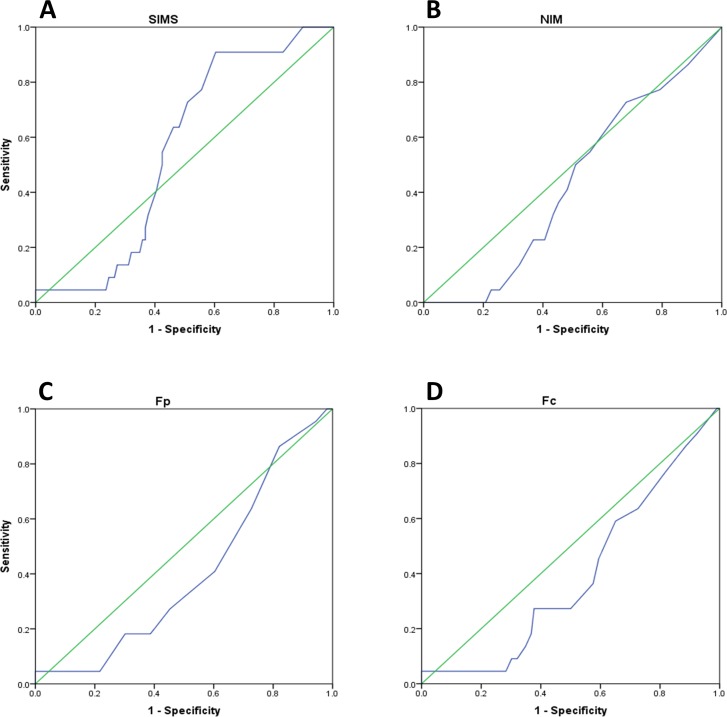
ROC curves for the Known-Group Comparison paradigm. (A) SIMS, (B) NIM, (C) Fp and (D) Fc Receiver Operating Characteristic curves (blue). The green diagonal in each panel indicates the reference value of 0.5 AUC (area under curve). For other abbreviations see text.

The sensitivity of SIMS in detecting suspected malingerers in the KGC design with respect to the other instruments was also shown by the Receiver Operating Characteristics (ROC) curves calculated for the four scales (SIMS, NIM, Fp and Fc) and presented in Table 5. Areas Under the Curve (AUC) values of KGC were in general much smaller than those of SD. However, SIMS AUC was the largest one among the four scales in KGC suggesting a better sensitivity of this measure with respect to the others. ROC curves for the KGC protocol are shown in Fig. 2.

**Table 5:** Areas under the Receiver Operating Characteristics Curve (AUC).

	KGC (*n* = 57)		SD (*n* = 71)	
	AUC	SEM	AUC	SEM
SIMS	0.553	0.055	0.940	0.022
NIM	0.433	0.058	0.926	0.031
Fp	0.404	0.061	0.771	0.056
Fc	0.392	0.060	0.836	0.045

**Notes.**

Values ± Standard Error of Mean (S.E.M.) are shown for the instruments tested in the two experimental designs, KGC and SD. AUC, area under curve; SEM, standard error of mean. For other abbreviations, see text.

Altogether, these findings show that SIMS was the only instrument among the evaluated ones characterized by a sufficient level of sensitivity to detect suspected malingerers in a KGC design and drove us to investigate the results of cut-off data (see Table 6).

**Table 6:** Percentage of participants detected by SIMS as malingerers and non-malingerers in the two experimental designs (cut-off >= 14).

KGC				SD			
Suspected malingerers		Controls		Simulated malingerers		Controls	
Nsim	Sim	Nsim	Sim	Nsim	Sim	Nsim	Sim
23%	77%	49%	51%	0%	100%	83%	17%

**Notes.**

Abbreviations as in Fig. 1.

As shown by Table 6, cut-off data were in line with the results of the ANOVA, qualitatively showing an excellent capability of SIMS in discriminating simulated malingerers from controls in the SD (100%). When looking at the KGC, however, the picture became much less clear. In fact, while 77% of suspected malingerers were detected as such, about one half of control participants (51%) were detected as malingerers as well. This observation testifies a quite high proportion of false positives in the population of psychiatric patients, already diagnosed before imprisonment and scoring above cutoff on the SCL-90-R. An alternative interpretation could be that the supposed sensitivity of SCL-90-R as an indicator of malingering (Sullivan & King, 2010), although not corroborated by the correlation study we showed in the present work, could have revealed a subpopulation of malingering individuals among those assumed to be genuine patients. Although unlikely (KGC_Controls were all diagnosed as psychiatric patients already before imprisonment), some exaggeration of symptoms remains possible making evident the necessity to proceed with caution when using a single instrument to detect psychiatric malingering in inmates populations.

## Discussion

The goal of the present study was to determine the effectiveness of three instruments used to detect malingering of psychiatric illness (Fp and Fc validity scales of the MMPI-2, NIM and SIMS) in a group of criminal defendants suspected of malingering because they manifested psychiatric symptoms only after imprisonment but scored negatively at the SCL-90-R (suspected malingerers, KGC_SM). As KGC_Controls we considered those inmates with a positive psychiatric diagnosis formulated both before and after imprisonment and positive at SCL-90-R (double convergence). All participants were requested to answer honestly to the tests. Moreover, because most of the tests have been validated by Simulation Design paradigms, we evaluated the same instruments by SD paradigm as well. To this purpose, we recruited inmates with negative psychiatric history and negative at the SCL-90-R. They were randomly assigned to two groups. The first group (simulated malingerers, SD_SM) included inmates asked to simulate psychiatric illness. The second group (SD_Controls) was formed by inmates requested to answer honestly to the tests. Results show that for the SD all the measures obtained high rates of accuracy in detecting malingering and were rather similar in terms of global performance indicators (ANOVA and AUC values). This finding is in agreement with what already reported in literature (for recent reviews on the use of SIMS in SD/KGC studies see Wisdom, Callahan & Shaw, 2010; Van Impelen et al., 2014). Conversely, for the KGC, three out of the four instruments showed poor performance in detecting malingering. ANOVA post-hoc analysis showed that only SIMS reached the significance threshold in discriminating KGC_SM from KGC_Controls.

While the effectiveness of the SIMS in detecting malingering in SD paradigms is largely confirmed by the literature (for an overview, see Edens, Otto & Dwyer, 1999), the case of KGC paradigms is much more complicated. On one side the number of studies using SIMS in KGC designs is much less than those based on SD (Wisdom, Callahan & Shaw, 2010; Van Impelen et al., 2014). On the other side, the criteria used to group individuals in KGC protocols often raise serious concerns. The most used criterion is the performance on the Structured Interview of Reported Symptoms (SIRS, Rogers, Bagby & Dickens, 1992; Edens, Poytress & Watkins-Clay, 2007; Sellbom et al., 2010). The SIRS uses multiple strategies to detect feigned psychopathology, such as absurd symptoms, unlikely combinations of symptoms, discrepancies between reported and observed symptoms and referred abnormal severity of symptoms. The SIRS has been well studied; a meta-analysis (Green & Rosenfeld, 2011) yielded a sensitivity (i.e., the likelihood of a positive symptom validity test, SVT result in feigners) of 0.49 and a specificity (i.e., likelihood of a negative SVT result in honest responders) of 0.95. The efficacy of SIRS in classifying KGC groups for the validation of the three tests we used in the present study has been shown for NIM (Rogers et al., 1998; Mogge & LePage, 2004; Bocaccini, Murrie & Duncan, 2006; Gaines et al., 2007), SIMS (Lewis, Simcox & Berry, 2002; Edens, Poytress & Watkins-Clay, 2007; Vitacco et al., 2007; Clegg, Fremouw & Mogge, 2009) and MMPI-2 (Bocaccini, Murrie & Duncan, 2006; Toomey, Kucharski & Duncan, 2009; Barber-Rioja et al., 2009). However, a strong concern on the use of SIRS to categorize participants in malingering-detecting KGC designs arises from the fact that it correlates with the majority of the other tests. Sellbom et al. (2010) reported a significant intercorrelation between SIRS and SIMS and between SIRS and NIM. Similar conclusions were reached by Laffon (2009) who also showed a correlation between SIRS and both, SIMS and NIM. Thus, the good sensitivity of SIRS in detecting malingering could arise from the fact that it was validated by using instruments strongly correlating with it. Moreover, according to Calhoun and coworkers (2000) SIRS seems to misclassify true patients as malingerers.

For these reasons, in our KGC paradigm we decided to use clinical criteria to create categories for the KGC design (psychiatric diagnosis and SCL-90-R) instead.

By doing this, we have been able to show that only the SIMS was able to discriminate suspected malingerers from controls. It should be stressed, however, that Edens, Otto & Dwyer (1999), found a positive correlation between SIMS and the GSI scale of the SCL-90-R and this argument could be used to generate a criticism similar to that we used for SIRS. However, the KGC group of suspected malingerers of our work was formed by individuals all scoring below the cutoff for SCL-90-R test. This should protects us from the criticisms that would arise from a situation similar to that of SIRS as underlined by Sellbom et al. (2010) and Laffon (2009). On the contrary, the absence of correlation between SCL-90-R and SIMS, NIM, Fp and Fc (r ranging from −0.12 to −0.14) seems to corroborate our approach.

In other words, we are aware of the difficulty in creating reliable known groups in a KGC design involving psychiatric inmates but in our study we used two criteria to extract a very specific subgroup of participants: inmates lacking any history of psychiatric diseases before incarceration and negative at the SCL-90-R test. According to these criteria, they were considered as ‘psychiatric patients’ only because of a diagnosis obtained after imprisonment. Such diagnoses are pretty easy to obtain because their main outcome, in general, is the administration of some anxiolytic or antidepressant medication (largely distributed in the prison environment and “appreciated” by both doctors and inmates for obvious practical reasons). Indeed, depression and mood-related disorders were the most represented in our population. One may argue that a further validation of our approach would have been provided by testing a further category in the KGC paradigm: inmates diagnosed by a psychiatrist only after imprisonment and scoring above threshold at the SCL-90-R test. In this case, no malingering behavior should be expected. In our view this would have only been a further confirmation of what we already report here. Moreover, the dimension of our sample did not allow the investigation of groups others than those described in the present study.

Rogers (1997a, 1997b) argued that the best approach to validate malingering-detecting psychometric tools is to compare validity measures obtained by both SD and KGC paradigms. Nevertheless, only few studies have been conducted using both SD and KGC on the same population (see Wisdom, Callahan & Shaw, 2010; Van Impelen et al., 2014). As far as we know, only one paper evaluated the efficacy of SIMS in detecting malingering in a group of defendants classified as supposed simulators by a psychological screening (Edens, Poytress & Watkins-Clay, 2007). According to this study, SIMS failed in detecting malingering. Here we found an opposite, positive result likely because of the different criteria used to categorize participants. At the same time, however, despite SIMS sensitivity, we showed a relatively poor specificity of this instrument (see the ROC analysis and the cutoff evaluation in our Results Section). We therefore agree with Edens, Poytress & Watkins-Clay (2007), when they say that SIMS specificity is pretty poor when administered to honestly responding, symptomatic individuals.

## Conclusions

Research to validate malingering-detecting tools is inherently problematic. Both over-identification and under-identification of feigning or exaggeration are reasons of concern. Despite our finding that SIMS significantly discriminates malingering defendants from controls in the KGC design, the poor specificity of this instrument lead us to conclude that one should be very cautious in using SIMS as a stand-alone measure. As far as we know this is one among the few studies where psychometric tools for malingering detection have been validated by both, Simulation Design and Known-Group Comparison. The main problem of Known-Group Comparison approaches is represented by the method adopted to create the experimental groups. Here we used both clinical and psychometric criteria and we are aware that the grouping criteria we decided *a priori* here are somewhat arbitrary. However, we are convinced that they could provide some useful cues to evaluate the solidity of malingering testing instruments outside the “protected environment” of the Simulation Design. Other works, on the contrary, have adopted psychometric instruments only (mainly the SIRS). Alternative grouping criteria as well as malingering behaviors in socio-demographic groups others than the European males evaluated by the present work should be explored by additional investigations. Finally, our results cannot be generalized to other populations often associated with a significant degree of malingering (e.g., personal injury litigants) different from criminal defendants. In conclusion, understanding what malingering looks like, how to design and use assessment measures and how to appropriately manage malingering behaviors remain therefore challenging questions which require responsible empirical approaches.

##  Supplemental Information

10.7717/peerj.5259/supp-1 Data S1 Raw data The individual scores obtained by participants on NIM, SIMS, MMPI_2 and SDCL-90-R tests. Participants are listed only by the experimental group they belong to. Variable labels are self-explanatory (see text).

## Additional Information and Declarations

### Data Availability

The following information was supplied regarding data availability: The raw data are provided in a Data S1.
